# Characteristics of Children with Diabetic Ketoacidosis Treated in Pediatric Intensive Care Unit: Two-Center Cross-Sectional Study in Croatia

**DOI:** 10.3390/medicina55070362

**Published:** 2019-07-10

**Authors:** Ivana Burcul, Nikolina Arambasic, Branka Polic, Tanja Kovacevic, Ines Bartulovic, Tatjana Catipovic Ardalic, Josko Markic

**Affiliations:** 1 Clinical Hospital Merkur, 10000 Zagreb, Croatia; 2 Department of Pediatrics, University Hospital of Osijek, 31000 Osijek, Croatia; 3 School of Medicine, University of Osijek, 31000 Osijek, Croatia; 4 Department of Pediatrics, University Hospital of Split, 21000 Split, Croatia; 5 School of Medicine, University of Split, 21000 Split, Croatia

**Keywords:** diabetic ketoacidosis, diabetes, children, cerebral edema, dehydration

## Abstract

*Background and objective:* There is an increasing risk of type 1 diabetes mellitus (T1D) among children in Croatia. Diabetic ketoacidosis (DKA) is the leading cause of morbidity and mortality in children with T1D, with cerebral edema as the most severe complication. Since early recognition of cerebral edema leads to a better outcome, it is important that patients with moderate or severe DKA are closely monitored and treated in pediatric intensive care units (PICUs). The aim of this study is to investigate clinical and laboratory parameters, as well as complications in children treated in PICUs because of DKA. *Materials and methods:* Patients treated due to DKA in the PICU of the University Hospitals of Split and Osijek from 2013 to 2017 were included in this study. Retrospectively collected data included age, gender, clinical signs and symptoms, and various laboratory parameters. After dividing subjects into two groups: Newly diagnosed with T1D (NT1D) and previously diagnosed with T1D (PT1D), collected data were compared between the two groups. *Results:* A total of 82 patients were enrolled. Those with NT1D were more often treated in the PICU, with two of them developing cerebral edema. Dehydration was the most frequent clinical sign, found in 95% of patients at admission. Decreased consciousness level was found in 41.5% of patients, with majority of them being somnolent. No difference was found between NT1D and PT1D. Additionally, there was no significant difference regarding laboratory data at admission. *Conclusions:* More children with NT1D required treatment in the PICU due to DKA with two of them developing cerebral edema. Since cerebral edema is a life-threatening condition, treatment of patients with moderate or severe DKA in PICUs will provide necessary monitoring enabling early recognition, treatment, and better treatment outcome. To minimize the incidence of DKA among patients with NT1D, it is important to continuously carry out public health education programs aimed at early identification of signs and symptoms of T1D.

## 1. Introduction

Type 1 diabetes mellitus (T1D) is one of the most common chronic diseases of childhood. In Croatia, there is an increasing incidence of T1D among children and adolescents, thus placing Croatia among the high-risk countries for T1D. A study published in 2014 showed that the incidence of T1D in Croatian children aged 0–14 over 2004–2012 was 17.23/100,000/year with an annual increase of 5.87% and is higher than the European average, which is 3.9% [1].

Although diabetic ketoacidosis (DKA) is not the most common acute complication of T1D in children and adolescents, it is the leading cause of morbidity and mortality [2]. DKA diagnosis is established according to the 2018 guidelines of the International Society for Pediatric and Adolescent Diabetes (ISPAD) [3]. Clinical manifestations of DKA include dehydration, tachypnea, Kussmaul breathing, acetone breath, nausea, vomiting, abdominal pain, confusion, drowsiness, progressive obtundation, and loss of consciousness. The severity of DKA is categorized by the degree of acidosis as mild, moderate, and severe. In mild DKA, the venous pH is less than 7.3 or serum bicarbonate is less than 15 mmol/L. In moderate DKA, pH is less than 7.2 and serum bicarbonate less than 10 mmol/L. In severe DKA, pH is less than 7.1 and serum bicarbonate less than 5 mmol/L [3].

The most severe complication of DKA is the development of cerebral edema, which is commonly mild and asymptomatic. Clinical, symptomatic cerebral edema, with a mortality rate of 20%–25% [4], is developed in approximately 0.5%–1% of children [5]. Since early recognition of cerebral edema leads to a better treatment outcome, pediatric intensive care unit (PICU) admission should be considered for all children who are at an increased risk for cerebral edema (<5 years of age, severe acidosis, high blood urea nitrogen (BUN), low partial carbon dioxide pressure) and for those with severe DKA (long duration of symptoms, compromised circulation, or depressed level of consciousness) [3]. Diagnostic criteria for cerebral edema include abnormal motor or verbal response to pain, decorticate or decerebrate posture, cranial nerve palsy, and abnormal respiratory pattern. Major criteria include altered mental status, bradycardia, and incontinence inappropriate for age. Minor criteria include vomiting, headache, lethargy, diastolic pressure >90 mmHg, and age <5 years [6].

The aim of this study is to investigate clinical and laboratory parameters in children treated in the PICU because of DKA, especially those with newly diagnosed T1D (NT1D), and to establish whether children with NT1D more often develop DKA requiring treatment in the PICU. Our intention is to investigate whether NT1D children have a higher risk of complications, most particularly cerebral edema, associated with DKA after the use of a standardized management protocol and to outline the importance of public health education in its prevention.

## 2. Materials and Methods

### 2.1. Participants

This retrospective cross-sectional study included patients aged 0 to 18 who were treated because of DKA in the PICU of the University Hospitals of Split and Osijek during the period from 1 January 2013 until 31 December 2017. These sites are two out of four Level III PICUs in Croatia. They cover almost half of the territory of Croatia and have the same indications for admission. The physicians in both centers have identical training backgrounds in pediatrics and intensive medicine and are using the same protocols for diagnosis as well for the treatment of DKA.

The study was approved by the University Hospital of Split Ethics Committees (No. 2181-147-01/06/M.S.-17-2) and the parents signed the informed consent.

### 2.2. Variables

Variables in this study included: (i) Variables obtained by standardized medical record, (ii) laboratory tests, (iii) clinical symptoms and signs, (iv) DKA diagnosis (observed as inclusive criterion), and (v) the complications associated with the treatment.

The medical record data included: age (in years), gender (male and female), body mass index (BMI; calculated by the standard equation: BMI = Body mass (kg)/Body height (m)^2^), personal history (i.e., celiac disease, autoimmune thyroiditis, goiter, obesity), and family history (parents, siblings, and grandparents) for diabetes.

Clinical symptoms and signs observed in this study included polydipsia, polyuria, nocturia, nausea, vomiting, abdominal pain, moderate to severe dehydration, Kussmaul breathing, acetone breath, and tachycardia (all observed at nominal/categorical scale: Yes/No).

DKA diagnosis was defined according to the criteria established by the ISPAD Consensus: Blood glucose >11 mmol/L, serum bicarbonate <15 mmol/L and/or venous pH < 7.3, ketonemia, or moderate or large ketonuria [3]. In this study we included only those patients who were admitted in the PICU.

The laboratory tests at the time of diagnosis included: Blood glucose, glycated hemoglobin (HbA1c), BUN, creatinine, sodium, potassium, pH, partial carbon dioxide pressure, serum bicarbonate, leucocytes, and hemoglobin. All variables obtained by laboratory tests were treated as continuous for the purpose of the statistical analyses (please see following text).

Additionally, the complications associated with the treatment (i.e., cerebral edema) were recorded. An analysis was performed among subgroups of newly diagnosed patients (NT1D) versus previously diagnosed ones (PT1D).

### 2.3. Statistics

Descriptive statistics were used to calculate all the data provided. The Kolmogorov–Smirnov test was used to evaluate the normality of the distributions of results. Consequently, the correlation of the continuous variables was tested by t-test for independent sample, while the Kruskal–Wallis test was used for ordinal variables. The differences between NT1D and PT1D for age-group distributions were calculated by Chi square test. The correlation of qualitative variables was tested by Fisher’s exact test. All calculations were done by SPSS statistical suite for Windows 23.0 (Armonk, NY, USA). Qualitative variables were described by percentages, while quantitative ones were described as mean and standard deviations. For all statistical calculations, the value *p* < 0.05 was considered statistically significant.

## 3. Results

In the study period, a total of 82 children (48/82 females, 58.5%) diagnosed with DKA were treated in the PICUs of the University Hospitals of Split and Osijek. Regarding the family history, T1D was found in 15.1% (11/73) and type 2 diabetes (T2D) in 32.9% (24/73) patients. Unknown type of diabetes in the family was reported in 4.1% (3/73) subjects, while 49.3% (36/73) of the patients had a negative family history for diabetes. The data for nine patients were missing. Personal history included celiac disease (5/82, 6.1%), autoimmune thyroiditis (4/82, 4.9%), goiter (2/82, 2.4%), and obesity (1/82, 1.2%).

Majority of the patients (57/82, 69.5%) were with NT1D. The average age in NT1D and PT1D groups were 9.9 ± 4.8 and 9 ± 5.6 years, with a BMI of 16.77 ± 3.75 and 17.32 ± 3.13 kg/m^2^, respectively. There were also no significant differences between the groups regarding the age group (Table 1).

Clinical characteristics of the patients are presented in Table 2. Except for nocturia, there is no significant difference between the groups.

**Figure 1:**
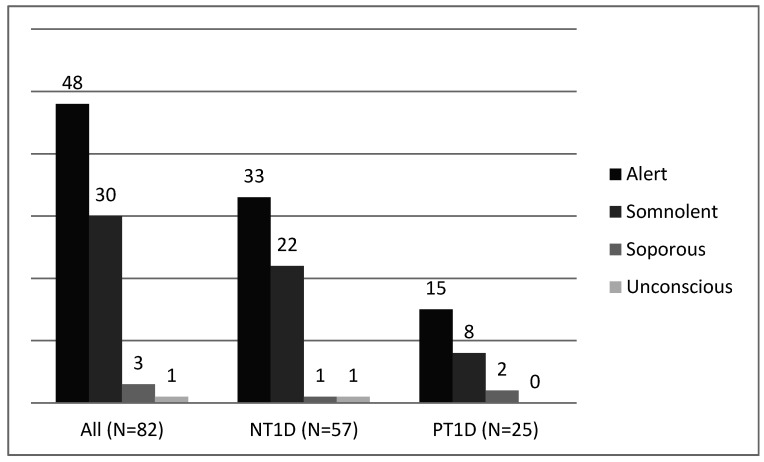
The level of consciousness of all (*n* = 82), NT1D (*n* = 57), and PT1D (*n* = 25) patients at admission. NT1D—newly diagnosed with type 1 diabetes mellitus; PT1D—previously diagnosed with type 1 diabetes mellitus.

The level of consciousness is presented in Figure 1. Out of all enrolled patients, 41.5% (34/82) had a decreased consciousness level at admission, with somnolence being the most prevalent one. We found no statistically significant difference between the NT1D and PT1D groups.

The significant difference between the groups was not found regarding the laboratory characteristics of the patients at admission, as well (Table 3).

Complications associated with the DKA were present in 4.9% (4/82) of patients. In the PT1D group, pneumomediastinum was noted in one child. In the NT1D group, three children had complications: Cerebral edema in one, cerebral edema together with acute pancreatitis in another, and the third one had sepsis caused by methicillin-sensitive *Staphylococcus aureus* (MSSA). Out of the total number of enrolled DKA patients, the rate of cerebral edema was 2.4% (2/82) with both of cases in the NT1D group.

## 4. Discussion

Cerebral edema is the most common cause of mortality in children with DKA and is associated with the development of neurological sequelae in the long term, as well. Other less common causes of mortality are attributable to severe infection or sepsis, pulmonary edema, acute respiratory distress syndrome, pneumomediastinum, hypo- or hyperkalemia, and cardiac arrhythmias [4]. Detection of decrease or deterioration in level of consciousness is essential in the diagnosis of cerebral edema [6]. In our study 41.5% of all included patients had decreased consciousness level with no statistically significant difference between the groups. Majority of them were somnolent. Three patients were soporous, while one patient in the NT1D group was unconscious. Lopes et al. found that 16.1% of NT1D patients and 9.5% of PT1D patients were somnolent at admission, which is a significantly lower percentage of patients in both groups compared to our study [7]. They also did not demonstrate statistically significant association between NT1D and PT1D related to the somnolence parameter. Nevertheless, in their research with 52 subjects they had a high percentage of children with cerebral edema. As many as 5.7% of their patients were treated for cerebral edema with no statistically significant difference in the incidence of cerebral edema between NT1D and PT1D [7].

In our study, two (2.4%) patients aged 10 and 11, both in the NT1D group, were diagnosed with cerebral edema. This considerably more than the world incidence of 0.5%–1% [5]. However, due to the small sample of patients in our research that relatively high percentage should be taken with reticence. Due to the possible development of cerebral edema, children with severe DKA including those with long duration of symptoms, compromised circulation or depressed level of consciousness, should be treated in the PICU, as well as those under five years of age, with severe acidosis, low partial carbon dioxide pressure, and high blood urea nitrogen [3]. Admission to the PICU enables meticulous monitoring of vital signs and clinical and neurological status by experienced staff allowing the recognition of early warning signs and symptoms of cerebral edema, timely adjustments in treatment and a better outcome. Clear written guidelines and access to laboratories for frequent evaluation of biochemical variables are also important.

In relation to the treatment, both our PICUs followed the same protocol for the management of DKA. In the initial phase, after the admission of the patient, shock and dehydration is treated with normal saline solution, followed by the use of intravenous insulin in continuous infusion avoiding boluses. Intravenous glucose is administered when more adequate blood glucose levels have been reached. Electrolyte balance should be preserved by adequate supply of sodium and potassium, contraindicating the use of sodium bicarbonate as part of the standard management.

Early detection of T1D is the key to preventing DKA. DKA usually reflects decreased awareness of the evolving symptoms of diabetes and its complications. One of the factors associated with a reduced risk of DKA at T1D diagnosis is a positive family history of diabetes [8]. Our findings did not reveal a significant impact of family history on the incidence of moderate or severe DKA treated in the PICU. Those results were similar to ones reported by Demir et al. who found a family history of T1DM and T2DM positive in 10.2% and 42.9% of the patients, respectively [9]. Medical care providers play a crucial role in the prevention of DKA as well since the risk of DKA increases significantly when diabetes is not diagnosed in a timely manner [10]. Therefore, a prevention campaign needs to spread the awareness of early symptoms of diabetes, which may not be initially distinguishable from other acute illnesses. Through educational campaigns it is possible to achieve and maintain a marked decrease in the incidence of DKA in children over five years old [11,12,13]. By providing posters to schools and providing local pediatricians with patient cards and equipment for measuring glucose, over the period the rate of DKA in children aged 6–14 years decreased to 12.5% compared with 83% in nearby areas. Moreover, the duration of symptoms was much shorter [11]. Iovane et al. suggest a new DKA prevention campaign aimed at children under five years of age [14]. Different authors showed that the age of onset of T1D has a bimodal distribution. The first peak was attributed to increased frequency of infections in the early school years and the second one is likely associated with pubertal changes. A recent Croatian study found that the first peak was in the age group between two and four years and was followed by a second peak in the 12–14 years age group [15]. In our study, the mean age among the total number of included children was 9.6 ± 5 years so we can conclude that the most common age group was pre-pubertal and pubertal. Therefore, educational programs need to be conducted at both primary and secondary schools targeting parents and teachers, as well as among general practitioners and pediatricians in primary care.

Precise determination of the hydration status is very important in the treatment of DKA. This study showed that 95% of children were diagnosed as moderately/severely dehydrated at admission. That can be attributed to usual symptoms of DKA including vomiting, which was the most common symptom at admission, and late arrival in the hospital. A study conducted at the Royal Children’s Hospital in Australia showed that the highest percentage of patients were dehydrated in ranges from 5% to 10% with a median of 8.7% [16]. Clinical assessment of the hydration status is often subjective, so up to 70% of patients may be overestimated or underestimated (24% and 46%, respectively) [16]. In order to make the clinical estimation of dehydration more objective, in moderate diabetic ketoacidosis it is recommended to estimate the loss of fluid to be 5%–7%, while it should be 7%–10% in severe DKA [3]. All PT1D children in our study had high HbA1c (11%–14%) due to poor control of their disease. The most common reasons for DKA in PT1D are insulin omission and, among insulin pump users, failure to take extra insulin with a pen or syringe when hyperglycemia occurs. Therefore, it is also of crucial importance to significantly increase effort in educating patients and their parents on how to manage diabetes and how to behave in specific situations. Furthermore, appropriate follow-up care by a diabetes team with a possibility of a 24-hour telephone helpline for emergency advice and treatment would be of importance [3].

DKA treatment has been widely studied; however, there are few studies comparing the characteristics of children admitted for DKA with NT1D and PT1D. The main strengths of our study are that we collected a significant number of patients and that we have analyzed the total number of them over a period of time, without exclusions that would constitute a bias in the analysis of the data; and that we have treated all cases under the same protocol. The limitation of the study is the fact that this was a retrospective study with a review of the medical data that were recorded by various doctors with different levels of expertise, especially regarding clinical characteristics at admission. An additional limitation is the fact that sample size was not calculated and that, with a bigger sample, significance could be reached. Additionally, since there is no Glasgow coma score in the medical records, there is a possibility of bias in the assessment of the consciousness level by different physicians.

## 5. Conclusions

This study showed that in two Croatian University Hospitals children with NT1D more often developed DKA requiring treatment in the PICU. They also showed a higher risk of developing cerebral edema as a complication of DKA and its treatment. Since cerebral edema is a life-threatening condition, treatment of patients with moderate or severe DKA in the PICU will provide necessary monitoring enabling the early recognition and treatment of cerebral edema and a better treatment outcome. Furthermore, to minimize the incidence of DKA among children with NT1D it is important to continuously carry out public health education programs aimed to facilitate early identification of signs and symptoms of T1D. Most cases of DKA could be prevented, as symptoms are usually present for several days or weeks before. For patients with PT1D, it is essential to educate and support both children and their families aiming for good control of diabetes and prevention of complications.

## Figures and Tables

**Table 1:** The age group distribution of the enrolled patients with differences between groups (Chi square *p* value presented; *n* = 82).

Age Group	NT1D *n* (%)	PT1D *n* (%)	*p* Value
0–5 years	12 (21)	8 (32)	0.899
6–10 years	16 (28.1)	5 (20)	0.224
11–15 years	24 (42.1)	9 (36)	0.800
16–18 years	5 (8.8)	3 (12)	0.178

NT1D—newly diagnosed with type 1 diabetes mellitus; PT1D—previously diagnosed with type 1 diabetes mellitus.

**Table 2:** Clinical characteristics of NT1D (*n* = 57) and PT1D (*n* = 25) patients at admission with differences between groups (*p* values for Fisher’s exact test presented).

Sign/Symptom	NT1D *n* (%)	PT1D *n* (%)	*p* Value
Polydipsia	27 (47.4)	14 (56)	0.81
Polyuria	23 (40.4)	14 (56)	0.465
Nocturia	16 (28.1)	1 (4)	0.008
Nausea	13 ( 22.8 )	6 ( 24 )	1
Vomiting	43 (75.4)	16 (64)	0.272
Abdominal pain	24 (42.1)	7 (28)	0.218
Moderate to severe dehydration	53 (93)	25 (100)	0.308
Kussmaul breathing	33 (58)	11 (44)	0.336
Acetone breath	38 (68)	16 (64)	0.801
Tachycardia	41 (72)	20 (80)	0.336

NT1D—newly diagnosed with type 1 diabetes mellitus; PT1D—previously diagnosed with type 1 diabetes mellitus.

**Table 3:** Laboratory characteristics of NT1D (*n* = 57) and PT1D (*n* = 25) patients at admission with differences between groups.

Laboratory Test	NT1D		PT1D		*T*-Test/Kruskal–Wallis Test		Reference Range
	Mean	SD	Mean	SD	t-Value/H	*p*	
Leukocytes, ×10^9^/L	19.28	10.69	19.66	9.61	0.28	0.59	4.4–11.6
Hemoglobin, g/L *	144.32	14.77	140.71	13.32	1.19	0.24	121–145
Glucose, mmol/L	31.35	12.12	29.38	7.22	0.07	0.78	3.9–5.9
HbA1c, %	11.10	1.87	11.35	1.76	1.4	0.23	<6
BUN, mmol/L	6.61	3.68	5.77	2.52	0.22	0.64	2.7–6.8
Creatinine, μmol/L *	75.9	44.23	72.36	46.31	0.27	0.79	37–63
Na, mmol/L	132.91	4.51	128.24	24.11	0.13	0.72	135–144
K, mmol/L	4.77	1.11	4.83	0.82	0.42	0.51	3.6–5
pH *	7.07	0.13	7.1	0.14	−0.89	0.38	7.35–7.45
pCO_2_, kPa	2.44	0.87	2.5	0.7	0.53	0.47	4.3–6
HCO_3_, mmol/L	9.27	17.95	6.64	3.54	1.1	0.29	18–23

SD—standard deviation; pCO_2_—partial carbon dioxide pressure; HCO_3_—serum bicarbonate; Na—sodium; K—potassium; HbA1c—glycated hemoglobin; BUN—blood urea nitrogen; NT1D—newly diagnosed with type 1 diabetes mellitus; PT1D—previously diagnosed with type 1 diabetes mellitus; * indicates variables where t-test was calculated due to normality of distribution.
